# Replacing Non-Active Video Gaming by Active Video Gaming to Prevent Excessive Weight Gain in Adolescents

**DOI:** 10.1371/journal.pone.0126023

**Published:** 2015-07-08

**Authors:** Monique Simons, Johannes Brug, Mai J. M. Chinapaw, Michiel de Boer, Jaap Seidell, Emely de Vet

**Affiliations:** 1 Department of Health Sciences and the EMGO Institute for Health and Care Research, Faculty of Earth and Life Sciences, VU University Amsterdam, The Netherlands; 2 Body@Work, Research Center Physical Activity, Work and Health, TNO- VU/VUmc, VU University Medical Center, Amsterdam, The Netherlands; 3 TNO, Expertise Centre Life Style, Leiden, The Netherlands; 4 Department of Epidemiology & Biostatistics and EMGO Institute for Health and Care Research, VU University Medical Center, Amsterdam, The Netherlands; 5 Department of Public and Occupational Health and the EMGO Institute for Health and Care Research, VU University Medical Center, Amsterdam, The Netherlands; 6 Chairgroup Strategic Communication, Sub-department Communication, Philosophy and Technology: Centre for Integrative Development, Wageningen University and Research Centre Wageningen, Wageningen, The Netherlands; NIDDK/NIH, UNITED STATES

## Abstract

**Objective:**

The aim of the current study was to evaluate the effects of and adherence to an active video game promotion intervention on anthropometrics, sedentary screen time and consumption of sugar-sweetened beverages and snacks among non-active video gaming adolescents who primarily were of healthy weight.

**Methods:**

We assigned 270 gaming (i.e. ≥2 hours/week non-active video game time) adolescents randomly to an intervention group (n = 140) (receiving active video games and encouragement to play) or a waiting-list control group (n = 130). BMI-SDS (SDS = adjusted for mean standard deviation score), waist circumference-SDS, hip circumference and sum of skinfolds were measured at baseline, at four and ten months follow-up (primary outcomes). Sedentary screen time, physical activity, consumption of sugar-sweetened beverages and snacks, and process measures (not at baseline) were assessed with self-reports at baseline, one, four and ten months follow-up. Multi-level-intention to treat-regression analyses were conducted.

**Results:**

The control group decreased significantly more than the intervention group on BMI-SDS (β = 0.074, 95%CI: 0.008;0.14), and sum of skinfolds (β = 3.22, 95%CI: 0.27;6.17) (overall effects). The intervention group had a significantly higher decrease in self-reported non-active video game time (β = -1.76, 95%CI: -3.20;-0.32) and total sedentary screen time (Exp (β = 0.81, 95%CI: 0.74;0.88) than the control group (overall effects). The process evaluation showed that 14% of the adolescents played the Move video games every week ≥1 hour/week during the whole intervention period.

**Conclusions:**

The active video game intervention did not result in lower values on anthropometrics in a group of ‘excessive’ non-active video gamers (mean ~ 14 hours/week) who primarily were of healthy weight compared to a control group throughout a ten-month-period. Even some effects in the unexpected direction were found, with the control group showing lower BMI-SDS and skin folds than the intervention group. The intervention did result in less self-reported sedentary screen time, although these results are likely biased by social desirability.

**Trial Registration:**

Dutch Trial Register NTR3228

## Introduction

Overweight and insufficient physical activity in youth are major public health concerns because of their associations with multiple chronic diseases [1–4]. Independent of physical activity, excessive sedentary time might also negatively affect health, although the evidence surrounding this issue is inconsistent [5,6]. The adolescent period is specifically characterized by a decline in physical activity [7,8], a high amount of sedentary screen time [9,10] and unfavorable changes in body composition (e.g. the amount and location of body fat) [11]. Adolescents are therefore an important target group for preventive interventions.

A common and popular activity among adolescents is playing video games [12–14]. Video games are often considered to be an important contributor to screen time and youth overweight [6,15,16]. However, video games are currently increasingly being explored as a means to promote physical activity and as a weight management tool [17–19]. Active video games,–i.e., video games that require physical activity to play-, can elicit light- to moderate-intensity physical activity [20,21] and might help to convert sedentary time into more active time. Studies have shown that many adolescents play active video games and enjoy playing them [12,22]. Hence, active video games might be capable of contributing to the prevention of excessive weight gain (i.e. weight gain that exceeds the weight gain required for regular growth).

In addition to increasing physical activity and decreasing sedentary behavior, active video gaming might have an additional effect on the prevention of overweight. Compared to non-active video gaming, active video gaming might provide fewer opportunities for snacking while gaming. In contrast active video gaming might also lead to increased intakes of snacks and beverages following the activity. The first studies that compared energy intake during active and non-active video gaming reported no differences [23,24]. However, these studies were controlled laboratory studies in adults [23] or used non-active video game performed on a treadmill for the active video game condition rather than a ‘real active video game’, such those played on the Wii, Kinect or PlayStation Move [24].

Thus far, there are a few large-scale randomized controlled trials (RCTs) that have evaluated the effects of active video games on body weight in adolescents [25]. Moreover, the majority of these studies focused on the treatment of overweight and obesity [26], included younger children [27,28] or evaluated active video games as part of a broader program [29]. Three large-scale RCTs have shown that active video gaming can beneficially affect body composition and overweight-related behaviors in overweight children [27–29]. However, the effects of providing active video games on the prevention of overweight in adolescents remain unclear.

The current trial aimed to evaluate whether providing active video games to non-active video gaming adolescents could contribute to the prevention of excessive weight gain. The adolescents were provided with the newest active video games to optimize engagement and an additional controller to facilitate the playing of the video games in multi-players modes. Furthermore, active video gaming was actively encouraged as a replacement for non-active video gaming.

We tested the hypotheses that four and ten months after randomization, the adolescents in the active video gaming intervention group would exhibit the following differences compared with the adolescents in the control group:

1. a lower body mass index adjusted for mean standard deviation score (BMI-SDS), a smaller waist circumference-SDS, a smaller hip circumference and a lower skin fold thickness
2. less self-reported time engaged in sedentary screen activities
3. lower self-reported intake of sugar-sweetened beverages and snacks.

To inform future active gaming studies and to gain additional more insight into the findings, a comprehensive process evaluation was conducted in the intervention group, to examine the following issues:

1. adherence to the intervention
2. appreciation of the Move video games and the intervention
3. game context
4. potential adverse effects (occurrence of injuries due to playing the Move video games)
5. activity replacement (i.e., activities that were replaced by active video gaming)
6. intention to continue playing the Move video games.

## Methods

### Design

The intervention was evaluated in a randomized controlled trial in adolescents aged 12- to 17-year-old adolescents. The study protocol has been described in detail in a separate publication [30]. The adolescents were randomly assigned to the intervention group or control group after baseline assessment by the researcher or a research assistant using a pre-determined computer-generated block randomization list with blocks of 100. The primary outcomes were the adolescents’ BMI-SDSs, waist circumference-SDSs, hip circumferences and skin fold thicknesses. Measurements of the primary outcomes were collected at baseline and after four and ten months. The secondary outcomes were the adolescents’ self-reported sedentary screen time and consumption of sugar-sweetened beverages and snacks. Additionally, physical activity behaviors excluding active video game play were assessed to determine whether the active video game intervention resulted in less traditional (non-electronic game) physical activity. Measurements of the secondary outcomes were collected at baseline and after one, four and ten months.

The study was approved by the Medical Ethics Committee of the VU Medical Centre. The trial is registered in the Dutch Trial Register with number NTR3228 (http://www.trialregister.nl/trialreg/admin/rctview.asp?TC=3228).

### Participants and recruitment

The current study focused primarily on adolescents aged 12–17 years. Additionally, the family members of the adolescent (i.e., parents and siblings aged 8–18 years) completed questionnaires, but these data were not included in the current analyses. The inclusion criteria were as follows:

- The adolescent played ≥ 2 hours of non-active video games per week.
- The adolescent played active video games less than once per week.
- The adolescent was physically and mentally able to play active video games (based on self-report).
- The adolescent had access to a PlayStation 3 at home.
- The family did not have a Move upgrade for the PlayStation 3.
- The adolescent lived in the same home as the participating family members at least 4 days per week (to enable sufficient access to the Move video games provided as part of the intervention, see below).
- At least one other family member (parent or sibling aged 8–18 years old) was willing to participate in the study (i.e., complete the questionnaires).

**Fig 1:**
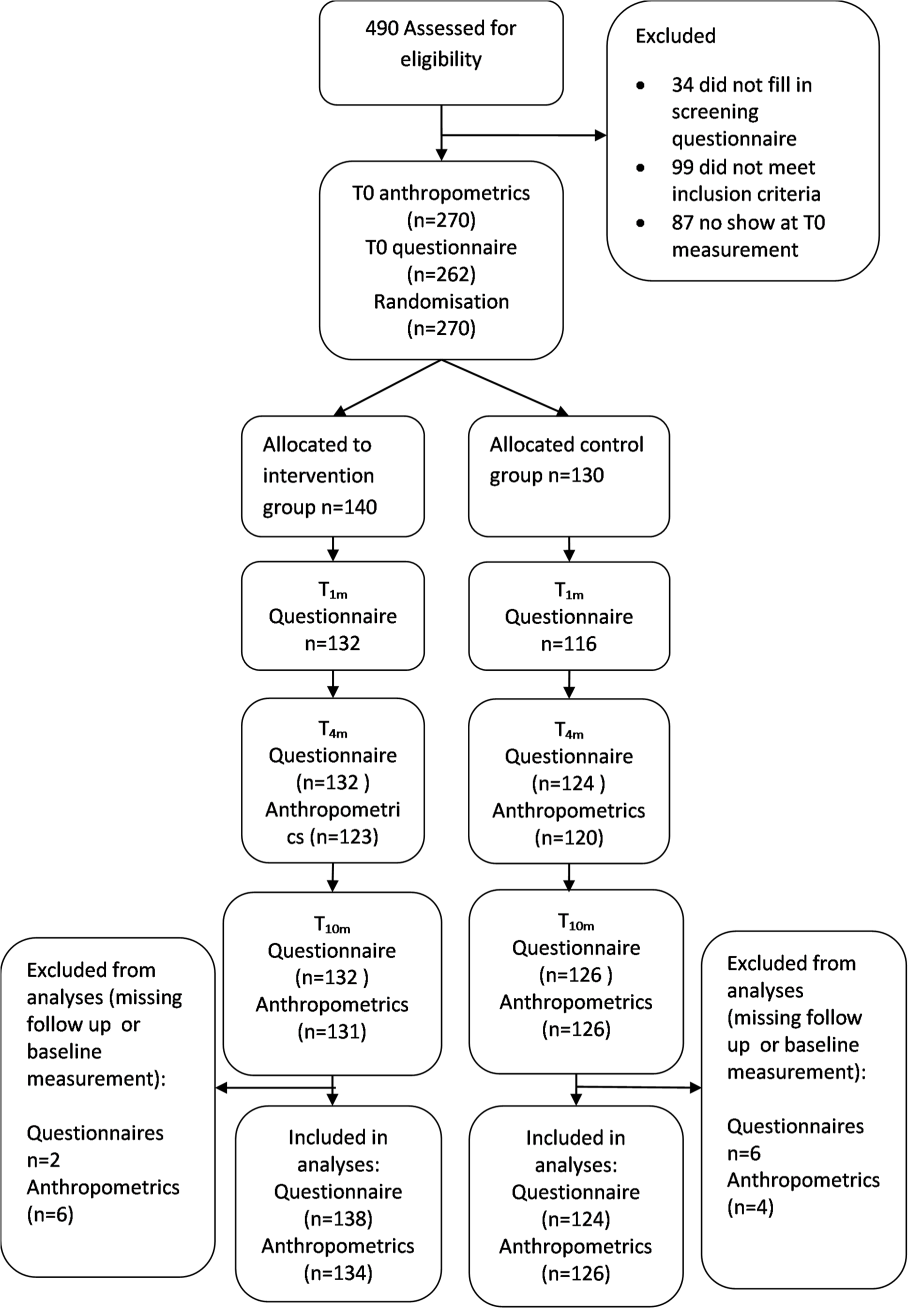
Participant flow chart.

The recruitment of the adolescents occurred in four cities in the Netherlands; i.e., Amsterdam, Amersfoort, Leiden and Breda. Detailed information about the recruitment is described in Simons et al. [30]. Adolescents and family members interested in participating provided their contact details on our project website or via e-mail and subsequently received an online screening questionnaire by email to assess their eligibility based on the inclusion criteria. We assessed 490 families for eligibility (see Fig 1 for a participant flow chart). The eligible families received information about participation that included a written consent form that the adolescents and their parents were required to complete prior to the collection of the baseline measurements. The consent procedure was approved by the ethics committee. Next, the, families received information about the baseline online questionnaires and were invited to appointments to provide the adolescent’s baseline measurements. Two hundred seventy adolescents showed up for the baseline measurements and were randomly allocated (140 to the intervention group and 130 to the control group). The sample size calculation (described in Simons et al. [30] indicated that we required 99 participants in each condition to have sufficient power to detect a clinically relevant difference in excessive weight gain of 0.5 kg (SD = 1.5 kg) between the intervention and control conditions during follow-up with a power of 0.80, alpha .05 and an intraclass correlation coefficient (ICC) for within-subject clustering of observations of 0.7. Based on an anticipated drop-out rate of 20%, a total of at least 119 adolescents per condition needed to be recruited. The 0.5 kg excessive weight gain was calculated based on adults, because calculations for adolescents were not available during the design of the study. On average adults gain 0.5 kg of excessive body weight per year due to an energy imbalance of 70 Kcal per week per year [31]. Based on energy expenditure studies, we calculated that an unnecessary weight gain of 0.5 kg might be prevented by substituting one hour per week of non-active video gaming with active video gaming [32]. In other words, we assumed that both groups would gain body weight but that the intervention group would gain 0.5 kg less than the control group due to the extra energy expenditure of playing the active video games.

### Intervention

The adolescents assigned to the intervention group received a PlayStation Move upgrade package to play the active video games on a PlayStation 3 console in their homes. The PlayStation Move uses a handheld motion controller wand, a motion-capture PlayStation Eye camera that tracks the player’s position and inertial sensors in the wand that detect its motion. Thus, every movement of the player is mimicked on-screen in the game. The following active video games were provided during the intervention: Sport Champions, Move Fitness, Start the Party and Medieval Moves, Dance Star Party and Sorcery. A detailed description of these Move video games can be found at: http://nl.playstation.com/ps3/games/.

Although, there are multiple active video game systems we chose for the PlayStation Move because the PlayStation appeared to be the most frequently owned video game console among 12–16 year old adolescents [12]. So by choosing an application that is compatible with the PlayStation, we optimized the chances for recruiting sufficient participants. Second, the active video game system for the PlayStation is one of the newest on the market at the time of the study, making use of the most sophisticated technology, and may be more affordable (around €50) than other consoles or active video game upgrades (e.g. Microsoft Kinect (around €135).

We included three elements to support continuing active video game play: 1) because variation in video games is important [22,33,34] the participants in the intervention group received four active Move video games with different game genres (Sport Champions, Move Fitness, Start the Party and Medieval Moves) at the beginning of the study and two additional video games (Dance Star Party and Sorcery) after four months; 2) because social and family play is important [22,35], we provided two controllers to promote playing together with family and friends; and 3) at each contact moment we explicitly asked and encouraged the participants to substitute non-active gaming with active gaming as much as possible and for at least one hour per week. One hour per week corresponds to approximately 70 kcal (which is equivalent to the energy imbalance that can result in unnecessary weight gain) [31] and was regarded as a feasible change [32].

Adolescents in the control group were asked to continue their normal gaming behavior. They received PlayStation Move starter packs at the end of the study as an incentive for their participation. Further, they received a small gift (e.g., a magazine, lanyard, or pen) as an incentive after participation at each measure moment.

### Procedures

The participants started in three waves for which baseline measurements were collected in January/February 2012, March 2012, and June 2012. The participants completed online questionnaires at baseline and at one, four and ten months of follow-up. Anthropometric measurements were collected at baseline and after four and ten months. Anthropometric measurements were conducted by trained research assistants according to a standardized protocol and occurred on pre-scheduled days at a central and attractive location (e.g., a museum or a soccer stadium). Adolescents who were unable to attend to central measurement location on the specified day were measured at home.

### Blinding

It was not possible to keep the participants blinded to the treatment allocation because the intervention group received an active video game upgrade package, and the control group did not. The participants and research assistants were blinded to group assignment at T_0_ but were not blinded atT_4m_ and T_10m_. The data analyses were not conducted in a blinded manner.

### Measurements

#### Adolescent anthropometrics (T_0_, T_4m_, T_10m_) (primary outcomes)

All measurements are described in detail in Simons et al. 2014 [30]. In short, we used a standardized measurement protocol to measure body weight, height, waist and hip circumferences and skinfold thickness (in the triceps, biceps, subscapular, and suprailiac regions) at T_0_, T_4m_, and T_10m_. BMI (kg/m^2^) was calculated by dividing the weight (kg) by the height squared (m^2^). Next, the BMI-SDS was determined using the data from the fourth Dutch growth study among children in 1997 as a reference [1] and employing the Growth Analyser software [36]. Regarding the waist circumference we also determined the SDS values using the same reference group and software employed for the BMI values. Regarding the hip circumference and skinfold thickness, valid Dutch reference data were not available for the calculations of SDS scores.

#### Self-reported measures

The questionnaires assessed demographics (i.e., birth date, sex, educational level (pre-vocational, higher continued education, or pre-university) and country of birth (to define ethnicity according the definition of Statistics Netherlands [37]). Furthermore, active and non-active gaming behaviors, other screen and physical activities, snack and sugar-sweetened beverage intakes, video game consoles/application owned and video game companions were assessed with questionnaires. Among the adolescents in the intervention group, a process evaluation was conducted by adding several process evaluation questions to the questionnaires that were administered at T_1m_, T_4m_, and T_10m_. Furthermore, the adolescents in the intervention group were asked to provide daily reports on their use of the Move video games over the entire ten-month period on a calendar. All of these self-reported measures are described in detail in Simons et al. [30]. We provide a summary of these measures below.

### Time spent on active and non-active video gaming (T_0_, T_1m_, T_4m_, T_10m_)

Questionnaires administered at T_0_, T_1m_, T_4m_, and T_10m_ assessed the total hours per week that were spent on active and non-active video games by asking about gaming frequencies and durations separately for week and weekend days.

### Physical activity and sedentary screen time (T_0_, T_1m_, T_4m_, T_10m_)

To assess physical activity, we used the validated (correlation with CSA: r = 0.48–0.78) Flemish Physical Activity Computerized Questionnaire (FPACQ) [38] and focused on the subdomains of active transport, leisure time walking and cycling and sports participation. The times spent in active transport, leisure time walking and cycling and sports participation were summed into a total physical activity score (hours per week).

To assess sedentary screen time, we used the questions about computer time and TV time from the FPACQ [38]. Both the computer and TV time were assessed separately for the week and weekend days and were then combined into total hours per week.

### Consumption of snacks and sugar sweetened beverages (T_0_, T_1m_, T_4m_, T_10m_)

Consumption of sugar-sweetened beverages was assessed based on the methods of Van der Horst et al. [39], which involve questions about the frequency and amount (numbers of glasses, cans and bottles) of carbonated and non-carbonated soft drinks, lemonade, and sports and energy drinks consumed on a typical day. Diet sodas and juices were not assessed. The total consumptions of sugar-sweetened beverages are expressed in milliliters per week.

The consumption of snacks was assessed using the appropriate items from a validated questionnaire [39,40]. Snacks were classified as savory (e.g., fast-food, pizza, fries, chips, and nuts) or sweet (e.g., candy, candy bars, chocolate, cake, and biscuits) foods eaten between the main meals and not as side servings at main meals. The quantity of consumed snacks was obtained by determining the number of ‘snack days’ and the amount of snacks consumed per ‘snack day’. These questions were combined into a single score to quantify the per-week snack intake.

### Game consoles and applications owned (T_0_, T_1m_, T_4m_, T_10m_)

At all measurements time points, we assessed the game consoles and game applications that the adolescents had access to in their homes to determine whether any participants in the control group purchased any active video game devices.

### Process evaluation measures

A comprehensive process evaluation was conducted in the intervention group at T_1m_, T_4m_ and T_10m_. Findings from focus groups [22] and a survey about active and non-active gaming in adolescents [12] provided rationales for evaluating the following six elements: 1) adherence to the intervention (usage of the Move video games and reasons for not playing at least one hour per week), 2) appreciation of the Move video games and the intervention (enjoyment, most played Move video game, most enjoying Move video game, ease of use, perceived competence, perceived physical exertion of playing Move video games, opinion about the number of provided Move video games, and self-purchase of Move video game), 3) game context (Move video game companions and location of the PlayStation Move console), 4) potential adverse effects (occurrence of injuries due to playing the Move video games), 5) activity replacement (the activities that were replaced by playing Move video games), and 6) intention to continue playing the Move video games.

In addition to the questionnaires, the adolescents in the intervention group reported the time that they spent playing the Move video games on a daily basis over the entire 10-month period in a calendar format. The adolescents were asked to report the type of Move video game and the number of hours and minutes spent playing the Move video games (cf. Chinapaw et al., 2008 [41]).

### Statistical analyses

The analyses were performed according the pre-defined analyses plan described in Simons et al. [30]. First, descriptive analyses were performed, and the data were examined for normal distributions. We report the medians and interquartile ranges of variables that were not normally distributed and the means and standard deviations of variable that were normally distributed. Total sedentary screen time was log transformed due to the non-normal distribution of this variable. The consumption of sugar-sweetened beverages was dichotomized into more or less than 1400 ml per week. The cut off value of 1400 ml per week was based on the recommendation of the Netherlands Nutrition Centre that no more than one sugar-sweetened beverage per day should be consumed [42] (one glass is approximately 200 ml x 7 days = 1400 ml per week). Subsequently, the control and intervention groups were described in terms of baseline demographics and outcome measures. Moreover, we analyzed whether there were any differences between the persons with complete outcome data and the persons with one or more missing data points at follow-up by performing Fischer’s exact, chi-square and t-tests separately for the anthropometric and questionnaire outcomes. Next, the effects of the intervention on all outcomes were evaluated by multilevel analyses with a random intercept on the person level. These models included the outcomes at one (only for the questionnaire outcomes), four and ten months and adjustments for the baseline values of the outcomes. For the continuous outcomes (i.e., BMI-SDS, waist circumference-SDS, hip circumference, skin fold thickness, non-active video game time, total sedentary screen time, physical activity and consumption of snacks), we used linear mixed models, whereas for the dichotomous outcomes (i.e., active video game time and consumption of sugar-sweetened beverages), we used logistic mixed models. In the main analyses, we analyzed the data from participants with at least a baseline value and one follow-up measurement. Ten persons had missing anthropometric data at follow-up, and eight persons had missing baseline questionnaire data; thus these persons could not be included in the main analyses. As sensitivity analyses for potential selection bias, we also imputed the first follow-up measurements for persons with only baseline anthropometric measurements (n = 10) and the baseline measurement for persons with missing baseline questionnaire data (n = 8) using multiple imputation with chained equations (true intention to treat). In the datasets with these imputed values (10 for the anthropometrics data and 8 for the questionnaire data), we then used the same mixed models. In addition to the model that was adjusted for baseline (model 1), we also constructed a second model that was additionally adjusted for demographics (age, sex, ethnicity and adolescent educational level) [1, 10, 43] (model 2).

Additionally, a group * time interaction was added to estimate the time-specific intervention effects. In the results section, only the fully adjusted models (model 2) are reported in the text. Model 1 is included in the tables as background information. Second, analyses were performed according to the per-protocol principle. Adherence to the protocol was separately defined for each follow-up measurement, based on the reported Move video game time in the questionnaire. The intervention group adolescents who played the Move video games at least one hour per week were allocated a score of 1 for adherence, and the intervention group adolescents who played less than one hour per week and the control group adolescents were scored 0. Again, two models were constructed for each outcome measure (with the exception of active video gaming) and were adjusted for the same variables as in the main analysis. Finally, descriptive statistics were used for the process evaluation measures and the use of the Move video games over the ten-month-intervention period based on the Move video game calendar.

Multiple imputation with chained equations and analyses of the imputed data were performed using STATA. Other statistical analyses were performed using IBM SPSS Statistics version 21 with a statistical significance level of p<0.05 for detecting main intervention effects.

## Results

### Participants

In total, 270 adolescents completed the anthropometric baseline measures and were randomly allocated to the intervention or control group. Of these 270 randomized adolescents, 260 participated in at least one of the anthropometric follow-up measurements and were included in the main analyses of the primary outcomes (anthropometrics). Two hundred sixty-two adolescents completed the baseline and at least one follow-up questionnaire and were included in the main analyses based on the questionnaire data (Fig 1). Missing data analyses revealed that the adolescents who missed one or more anthropometric measurements (n = 30) did not differ in age (T(268): -1.296; *P* = 0.19), sex (Fisher’s exact *P* = 0.51), educational level (Fisher’s exact *P* = 0.392) or ethnicity (Fisher’s exact *P* = 0.620) from the adolescents with complete anthropometric data (n = 240). Further, the adolescents who missed one or more questionnaire measurement (n = 42) did not differ in age (T(268): -1.235; *P* = 0.218), sex (Fisher’s exact *P* = 1.0), educational level (Fisher’s exact *P* = 0.064) or ethnicity (Fisher’s exact *P* = 0.074) from the adolescents with complete questionnaire data (n = 228).

Table 1 shows the baseline characteristics of the adolescents. The mean age was 13.9 (SD = 1.3) years, the majorities were boys, engaged in higher levels of secondary education, and of Dutch ethnicity, and approximately 1/4 were overweight or obese according the international cut off value of Cole et al., 2000 [44].

**Table 1:** Baseline characteristics of adolescent study participants (mean (SD) or %).

	Intervention group	Control group	Total
***Demographics***	N = 134	N = 126	N = 260
Age, mean (SD)	13.7 (1.3)	14.1 (1.3)	13.9 (1.3)
Sex, % boys	90	92	91
Educational level, % following higher educational level^a^	72	65	69
Ethnicity, % Dutch origin^b^	85	80	83
***Anthropometrics***			
BMI	20.6 (3.7)	20.3 (3.0)	20.5 (3.4)
BMI-SDS^c^	0.48 (1.2)	0.35 (1.1)	0.42 (1.1)
% Overweight/ obese^d^	25	19	22
SDS-waist circumference^c^	0.53 (1.07)	0.36 (0.98)	0.45 (1.03)
Hip circumference	88.4 (8.4)	87.9 (7.7)	88.2 (8.04)
Sum of skin folds	52.1 (31.3)	50.0 (26.5)	51.1 (29.01)
***Behavior***	N = 138	N = 124	N = 262
Active video gaming (hours per week),			
% ≥ 1 hour per week	21	29	25
Non-active video gaming (hours per week),			
median (IQR)	12.0 (11.0)	9.58 (10.81)	11.0 (10.7)
Total sedentary screen time (hours per week),			
median (IQR)	39.25 (28.0)	36.33 (20.98)	37.42 (24.23)
Physical activity^e^ (h/week),			
median (IQR)	10.63 (7.02)	10.38 (6.42)	10.5 (7.0)
Snack intake (pieces/portions per week),			
median (IQR)	10.0 (12.25)	12.0 (13.0)	11.5 (13.0)
Consumption of sugar sweetened beverages,			
% >1400 ml/week	73	76	74

^a^Higher educational level = higher continued education and Pre-university education (low educational level = pre-vocational education).

^b^Adolescents were defined as Dutch origin when both parents were born in the Netherlands (CBS).

^c^SDS scores were calculated with the use of data from 1997 described by Schönbeck et al., 2011 [1].

^d^Based on international cut-off points for overweight and obesity (Cole et al., 2000) [44].

^e^Sum of active transport to school, walking and cycling for transport in leisure time and sports participation.

IQR = Interquartile Range.

### Effects on anthropometric parameters

Table 2 presents the descriptive information for all of the anthropometric outcomes at baseline, T_4m_, and T_10m_ for the intervention and control group and the results of the main multilevel analyses. In the fully adjusted analyses (model 2), we observed significant intervention effects in the unintended direction at T_10m_ and overall for BMI-SDS and the sum of the skin folds. Regarding hip circumference, we found a time x group interaction effect (*P* = 0.015); there was no significant difference at T_4m_, but at T_10m_, hip circumference was significantly higher in the intervention group than in the control group. We observed no significant intervention effects on waist circumference-SDS in the fully adjusted models. The sensitivity analyses on the imputed data produced very similar results (not reported here).

**Table 2:** Results of main multilevel regression analyses (β (95% CI)) to evaluate the effects of the active game intervention on anthropometrics after 4 and 10 months (statistical significant β with their 95%CI’s are printed in bold).

		Intervention group		Control group	Model 1^b^	Model 2^c^
	N	Mean (SD)	N	Mean (SD)	β (95%CI)	β (95%CI)
BMI-SDS^a^						
Baseline	134	0.48 (1.2)	126	0.35 (1.1)		
4-months	123	0.51 (1.2)	120	0.33 (1.0)	0.044 (-0.035; 0.123)	0.049 (-0.031;0.128)
10-months	131	0.49 (1.1)	126	0.28 (1.0)	0.093 (0.015; 0.17)	0.098 (0.0199;0.176)
*Overall effect*					0.069 (0.003;0.135)	0.074 (0.008;0.14)
SDS-waist circumference^a^						
Baseline	134	0.53 (1.07)	126	0.36 (0.98)		
4-months	123	0.61 (1.04)	120	0.45 (1.0)	0.05 (-0.246;0.347)	0.025 (-0.271;0.321)
10-months	131	0.63 (1.05)	126	0.37 (0.98)	0.258 (0.010;0.506)	0.23 (-0.017;0.476)
*Overall effect*					0.045 (-0.043;0.132)	0.039 (-0.046;0.125)
Hip circumference (cm)						
Baseline	134	88.4 (8.4)	126	87.9 (7.7)		
4-months	123	89.3 (8.0)	120	89.1 (7.4)	0.025(-0.615;0.665)	0.004(-0.645;0.653)
10-months	131	89.8 (7.9)	126	88.5 (6.9)	0.823(0.196;1,451)	0.751(0.113;1.39)
*Overall effect*					0.44 (-0.121;1.00)	0.39 (-0.18;0.964)
Sum of skin folds (mm)						
Baseline	134	52.1 (31.3)	126	50.0 (26.5)		
4-months	123	51.1 (28.9)	120	46.5 (24.5)	2.27 (-2.94;7.48)	2.50 (-2.75;7.76)
10-months	131	50.0 (31.6)	126	44.7 (23.7)	3.42 (0.227;6.609)	3.45 (0.196;6.71)
*Overall effect*					3.13 (0.246;6.019)	3.22 (0.266;6.17)

^a^SDS-BMI–standard deviation of body mass index; SDS-waist circumference–standard deviation of waist circumference.

^b^Model 1 adjusted for baseline outcome value.

^c^Model 2 adjusted for baseline outcome value, age, sex, ethnicity and adolescent educational level.

Control group = 0; Intervention group = 1 (ref).

The per-protocol analyses revealed no significant overall effects of adherence to the protocol (i.e., playing the Move video games for at least one hour per week) on BMI-SDS (Model 1: β = 0.048, 95% confidence Interval (CI): -0.029 to 0.126; Model 2: β = -0.05, 95% CI: -0.186 to 0.084), waist circumference-SDS (Model 1: B = 0.0896, 95% CI: -0.012 to 0.192; Model 2: β = 0.085, 95% CI: -0.0146 to 0.185), or hip circumference (Model 1: β = 0.042, 95% CI: -0.577 to 0.662; Model 2: β = 0.0089, 95% CI: -0.608 to 0.625) and a significant effect in the unintended direction on the sum of the skinfolds (Model 1: β = 3.28, 95% CI: 0.211 to 6.355; Model 2: β = 3.11, 95% CI: 0.04 to 6.18).

### Effects on behaviors

Table 3 presents the descriptive information about the behavioral outcomes at baseline, T_1m_, T_4m_, and T_10m_ for the intervention and control groups and the results of the multilevel analyses. Based on the fully adjusted models (model 2), the intervention group was more likely to play active video games for at least one hour per week. Regarding active video gaming we observed a time x group interaction effect (*P* = 0.015) in which the effect diminished over time (Table 3). Further, the intervention had a beneficial overall effect on the time spent playing non-active video games in favor of the intervention group. Additionally, overall, the intervention group exhibited a 0.8 fold reduction in the geometric mean of sedentary screen time (i.e., TV/DVD, non-active video games, and computer time combined) compared to the control group (Table 3). Regarding the consumption of sugar-sweetened beverages, total physical activity and the consumption of snacks, we found no significant differences based on the fully adjusted model (model 2). The sensitivity analyses on the imputed data produced very similar results (not reported here).

**Table 3:** Results of intention to treat multilevel regression analysis (β (95% CI)) to evaluate the effects of the active video game intervention on video game behavior, sedentary screen time, physical activity and energy intake after 1, 4 and 10 months (statistical significant β with their 95%CI’s are printed in bold).

		Intervention group		Control group		Model 1^a^	Model 2^b^
	N		N				
**Active video game time (≥ 1 hours per week) (yes = 1; no = 0)**		% ≥ 1 hour per week				**OR**	**OR**
Baseline	138	21	124				
1-month	131	94	111			**133.98 (48.25; 372.06)**	**126.79 (44.59; 360.56)**
4-months	130	85	119			**54.94 (23.03; 131.05)**	**57.52 (23.38; 141.5)**
10-months	131	78	121			**27.33 (12.21;61.16)**	**29.34 (12.70; 67.75)**
*Overall effect*						**48.3 (26.84; 86.95)**	**49.47 (26.8; 91.29)**
**Non-active video game time (hours per week)**		**Median (IQR)**		**(Median (IQR)**		**β (95%CI)**	**β (95%CI)**
Baseline	138	12.0 (11.0)	124	9.58 (10.81)			
1-month	131	8.7 (9.0)	111	10.7 (9.0)		**-1.9 (-3.72;-0.089)**	**-1.49 (-3.33;0.35)**
4-months	130	6.6 (7.6)	119	9.0 (7.5)		**-1.78 (-3.58;0.018)**	**-1.33 (-3.15;0.48)**
10-months	131	6.8 (6.0)	121	8.3 (9.8)		**-2.94 (-4.74;-1.15)**	**-2.49 (-4.31;-0.67)**
*Overall effect*						**-2.2 (-3.63;-0.78)**	**-1.76 (-3.20; -0.32)**
**Total sedentary screen time (hours per week)** ^**c**^		**Median (IQR)**		**Median (IQR)**		**Exp (β) (95%CI)** ^**e**^	**Exp (β) (95%CI)** ^**e**^
Baseline	138	39.25 (28.0)	122	36.33 (20.98)			
1-month	130	31.5 (25.35)	110	38.71 (23.58)		**0.78 (0.70;0.86)**	**0.82 (0.73;0.91)**
4-months	129	29.0 (19.88)	119	35.0 (23.22)		**0.82 (0.74;0.90)**	**0.78 (0.69;0.87)**
10-months	131	30.5 (22.0)	121	34.83 (23.70)		**0.79 (0.72;0.88)**	**0.82 (0.74;0.92)**
*Overall effect*						**0.80 (0.72; 0.86)**	**0.81 (0.74; 0.88)**
**Physical activity** ^**d**^ **(hours per week)**		**Median (IQR)**		**Median (IQR)**		**β (95%CI)**	**β (95%CI)**
Baseline	138	10.63 (7.02)	124	10.38 (6.42)			
1-month	131	10.17 (6.17)	111	10.36 (6.33)		-0.24 (-1.34;0.86)	-0.40 (-1.53;0.73)
4-months	130	10.25 (5.92)	119	10.25 (6.33)		-0.05 (-1.15;1.04)	-0.56 (-1.72;0.59)
10-months	131	10.0 (6.17)	121	10.0 (6.96)		-0.08 (-1.17;1.01)	-0.37 (-1.5;0.77)
*Overall effect*						-0.12 (-1.04;0.80)	-0.43 (-1.34;0.48)
**Consumption of sugar-sweetened beverages (>1400 ml per week (yes = 1; no = 0)**		**% >1400 ml/week**	**%>1400ml/week**			**OR (95%CI)**	**OR (95%CI)**
Baseline	138	73	124		76		
1-month	131	61	111		78	**0.50 (0.25;0.98)**	0.49 (0.24;1.01)
4-months	130	60	119		71	0.69 (0.36;1.33)	0.74 (0.38;1.47)
10-months	131	62	121		77	0.67 (0.34;1.29)	0.71 (0.36;1.41)
*Overall effect*						**0.62 (0.40; 0.96)**	0.65 (0.41;1.03)
**Consumption of snacks (pieces/portions per week)**		**Median (IQR)**			**Median (IQR)**		
Baseline	138	10.0 (12.25)	124		12.0 (13.0)		
1-month	131	10.0 (11.0)	111		11.0 (11.0)	-1.44 (-3.38;0.50)	-0.98 (-3.03;1.07)
4-months	130	9.0 (9.0)	119		10.0 (12.0)	-0.72 (-2.64;1.20)	-1.76 (-3.86;0.33)
10-months	131	9.0 (11.0)	121		11.0 (10.5)	-1.11 (-3.03;0.82)	-0.87 (-2.93;1.18)
*Overall effect*						-1.07 (-2.66;0.50)	-1.12 (-2.75;0.50)

Control group = 0 (reference group); Intervention group = 1.

^a^Model 1 adjusted for baseline outcome value.

^b^Model 2 adjusted for baseline outcome value, age, sex, ethnicity and adolescent educational level.

^c^Total sedentary screen time includes TV/DVD, non-active game and computer time.

^d^Physical activity includes sports, active transport to school and walking and cycling in leisure time.

^e^Value represents Exp (β) (95%CI) which is the ratio of geometrical means.

IQR = Interquartile Range.

The per-protocol analyses revealed no significant overall effects of adherence to the protocol (i.e., playing the Move video games for at least one hour per week) on non-active gaming (Model 1: β = -0.34, 95% CI: 0–1.78 to 1.09; Model 2: β = -0.09, 95% CI: -1.52 to 1.34), total sedentary screen time (Model 1: Exp (β) (ratio of geometrical mean) = 0.94, 95% CI: 0.87 to 1.03; Model 2: Exp (β) = 0.96, 95% CI: 0.88 to 1.05), physical activity (Model 1: β = -0.28 95% CI: -1.11 to 0.55; Model 2: β = -0.29, 95% CI: -1.11 to 0.53), consumption of sugar-sweetened beverages (Model 1: OR = 1.1, 95% CI: 0.68 to 1.79; Model 2: OR = 1.36, 95% CI: 0.82 to 2.27), or consumption of snacks (Model 1: β = -0.15, 95% CI: -1.65 to 1.35; Model 2: β = -0.11, 95% CI: -1.62 to 1.41).

### Process evaluation

Table 4 shows the results of the process evaluation. At T_1m_, slightly more than half of the adolescents in the intervention group reported playing the Move video games for at least one hour per week, and after ten months, this proportion was approximately one-third. Across the entire intervention period, 14% of the adolescents reported playing the Move video games every week for at least one hour per week at all the time points.

**Table 4:** Process evaluation outcome measures at 1 month, 4 months and 10 months.

	1 month	4 months	10 months
*1*. *Adherence to intervention*			
How much time did you spend on average playing the Move video games? (% (n))			
0–60 minutes per week	42 (54)	60 (79)	67 (87)
≥60 minutes per week	58 (74)	40 (51)	33 (44)
Did you succeed in playing the move video games for at least one hour per week?			
Yes, I played the move games for at least one hour per week	61 (79)	33 (43)	28 (37)
No, in some weeks I failed to play the move games for at least one hour	37 (48)	58 (77)	55 (73)
No, I never succeed in playing the move games for at least one hour per week	2 (3)	9 (12)	17 (22)
Reasons for not playing the Move games among adolescents who did not succeed to play ≥ 1 hour per week Move games (top 5) (% (N))			
Lack of time	64 (32)	67 (59)	71 (67)
Too many other things to do	56 (28)	66 (58)	65 (61)
I rather play non-active video games	24 (12)	42 (37)	50 (47)
Move games were boring	18 (9)	30 (26)	37 (35)
Lack of space to play the Move games	22 (11)	18 (16)	16 (15)
*2*. *Appreciation of the Move games and the intervention*			
Enjoyment (scale 1–7) (mean (SD))	4.8 (1.2)	4.0 (1.4)	3.7 (1.5)
Ease of use (scale 1–5) (mean (SD))	4.4 (0.7)	4.4 (0.7)	4.3 (0.7)
Competence (scale 1–5) (mean (SD))	3.7 (0.6)	3.6 (0.6)	3.5 (0.7)
Most often played Move game (based on a rank score from 1 to 6) (mean (SD))			
Dance start Party	-	-	4.2 (1.7)
Start the Party: Save the World	-	-	4.2 (1.7)
Sorcery	-	-	3.7 (1.7)
Move Fitness	-	-	3.5 (1.5)
Medieval Moves	-	-	3.3 (1.3)
Sports Champions	-	-	2.1 (1.4)
Most enjoyed Move games (based on the mean rank score (1 to 6) (Mean (SD))			
Start the Party: Save the World	-	-	4.1 (1.7)
DanceStar Party	-	-	4.1 (1.7)
Sorcey	-	-	3.6 (1.8)
Move Fitness	-	-	3.6 (1.5)
Medieval Moves	-	-	3.5 (1.4)
Sports Champions	-	-	2.2 (1.4)
Opinion on the amount of provided Move games (% (n))			
Too little	-	-	21 (27)
Good	-	-	74 (98)
Too much	-	-	5 (6)
Did you bought, received, borrowed or downloaded other Move games in addition to the games we provided you? (% (N) yes)	-	-	26 (34)
Perceived physical exertion of playing Move games (% (n))			
Light	-	-	37 (49)
Moderate	-	-	58 (75)
Heavy	-	-	5 (7)
*3*. *Game context*			
Move game companion (% (n))			
Alone	-	-	58 (77)
With friends	-	-	21 (27)
With brother/sister	-	-	16 (21)
With others	-	-	3 (4)
With my parents	-	-	2 (2)
Location of PlayStation Move console			
Living room	-	-	42 (55)
Adolescent’s bed room	-	-	42 (55)
Brother/sister’s bedroom	-	-	6 (8)
Attic	-	-	5 (7)
Shared game/computer room	-	-	5 (6)
*4*. *Potential adverse effects*			
Injuries (bruises or strained muscles/tendons) while playing Move video games (% (N))	-	-	20% (26)
*5*. *Activity replacement*			
Playing Move video games mainly replaced ….			
Playing non-active game	-	-	65 (85)
Watching TV	-	-	11 (14)
Surfing the web	-	-	8 (11)
Sports	-	-	4 (5)
Playing outside	-	-	2 (2)
Other …	-	-	10 (14)
*6*. *Intention to continue playing the Move video games*			
I intend to continue playing the Move video games (% (N) agree or totally agree)	-	-	44 (57)
I expect to continue with playing the Move video games (totally disagree (% (N) probably or very probably)	-	-	49 (64)

**Fig 2:**
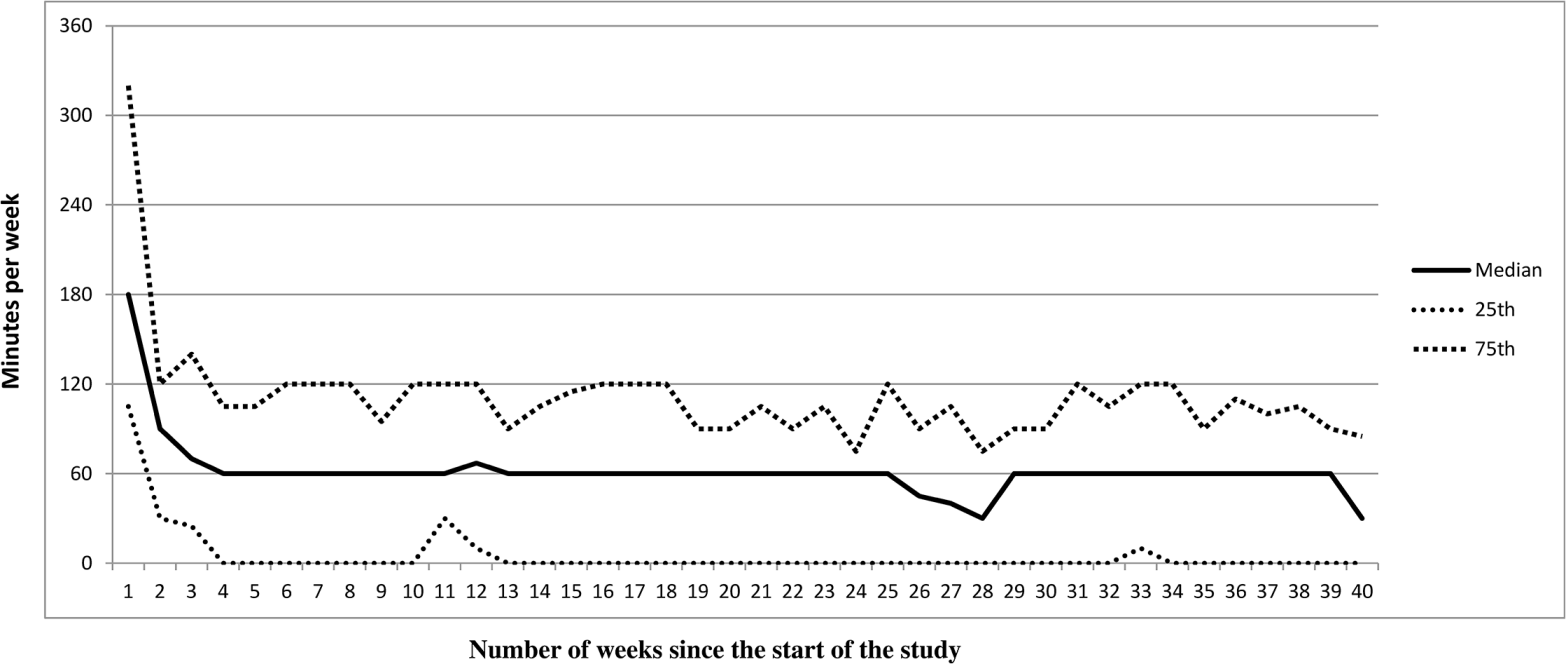
Development of usage of the Move games (minutes per week) over the 10-month (40 weeks) intervention period.

Fig 2 shows the development of time spent playing the Move video games (minutes per week) over the 40-week-intervention period based on the Move game calendars. After a peak in the first week, the median declined to approximately 60 minutes per week. From week 4 onwards, the 25th percentile was generally on the null line, indicating that at least 25% of the participants did not play the Move games at all during most of the intervention period. The adolescents who did not always play the Move video games for the minimum of one hour per week (N = 50) indicated that a lack of time or too many other things to do were the main reasons for not meeting this minimum. ‘I Rather played non-active video games’ was increasingly mentioned a reason during the course of the intervention. Further, the adolescents increasingly reported that the Move video games were boring.

The mean enjoyment score was moderately positive and exhibited a decreasing trend over the intervention period. The participants thought that the Move video games were easy to handle based on the Ease of Use scores. Furthermore, the participants thought that they were moderately competent in playing the Move video games. The most-often played and most enjoyed Move video games were DanceStar Party and Start the Party: Save the World. The least played Move video game was Sports Champions. Most of the adolescents were satisfied with the number of Move video games provided. Further, approximately 1/4 of the adolescents bought extra Move video games themselves (e.g. Killzone 3, FIFA 13, Just Dance, Little Big Planet 2, Virtua Tennis 4, and Top Spin). Further, the majority of the adolescents perceived playing the Move video games to be a moderate-intense activity. Regarding game context, the majority of the adolescents played the Move video games alone, and most placed the PlayStation Move in the living room or in their own bedroom.

At T_10m_, 1/5 of the intervention group reported having experienced an injury (the most frequently mentioned injuries were bruises or strained muscles/tendons) while playing the Move video games. The majority of the adolescents reported that the Move video games primarily replaced non-active video games. Finally, nearly half of the adolescents intended to continue playing with the Move video games.

One adolescent in the control group reported having a PlayStation Move at baseline but not during the follow-up measurements. At T_10m_ one additional adolescent in the control group reported having a PlayStation Move application.

## Discussion and Conclusion

The aim of the current study was to evaluate whether providing active video games could prevent unnecessary increases in anthropometrics among gaming adolescents who were primarily of healthy weight. The findings regarding the anthropometrics revealed that the active video game intervention had no effects in favor of the intervention group and even some effects in the unexpected direction were observed. The intervention group remained relatively stable regarding most of the anthropometric outcomes over time, and the control group improved somewhat in terms of their anthropometric outcomes. Furthermore, some beneficial effects on self-reported non-active gaming and total sedentary screen time (i.e., watching TV/DVD, computer time and non-active gaming combined) were found in favor of the intervention group. We found that the intervention had no effects, either positive or negative, on the levels of self-reported physical activity or the intakes of sugar-sweetened beverages and snacks. Compliance with the intervention was very low, although the adolescents in the intervention group were significantly more likely to play the active video games for at least one hour per week than the adolescents in the control group (OR = 49). It is important to acknowledge that the ORs for active gaming (and also for the consumption of sugar-sweetened beverages) should not be interpreted as risk ratios due to the high proportion of the sample who reported that they engaged in active gaming for at least one hour per week (or consumed >1400 ml/week sugar-sweetened beverages) at each time point.

Unexpectedly, the control group exhibited improvements in BMI-SDS, while the intervention group generally remained stable. The process evaluation did not provide any reason to believe that the adolescents in the control group bought Move packages themselves and began playing (more) active video games. Further, no evidence was found that indicated meaningful differences in life style behaviors between the intervention and control group. Although the intervention aimed to enable and motivate the participants to engage in active video gaming, only 14% of the participants in the intervention group managed to play for at least one hour per week throughout the intervention period. The dose of active video game play might thus have been insufficient to induce differences in the anthropometrics between the intervention and the control group. A second reason for the lack of an effect on the BMI-SDS of the intervention group might be that our study focused on a general group of gaming adolescents who primarily were of healthy weight. In accordance with our study’s purpose,–i.e., to evaluate the role of active gaming in the primary prevention of overweight and obesity in youth,- we therefore did not select a high-risk group such as adolescents who were already overweight or obese. Earlier studies that focused on overweight children, and thus more on the ‘treatment’ of overweight, have reported beneficial effects on BMI and/or body composition [26,27,29]. These findings suggest that active video games might be more effective in higher-risk populations such as overweight adolescents. A third reason for the lack of effects might be that our intervention aimed to replace non-active gaming with active gaming and thus attempted to change non-active video gamers into active video gamers. Therefore, we recruited adolescents who spend substantial amounts of time on non-active gaming. At baseline, our participants spent on average almost 14 hours per week playing non-active games. Based on other time expenditure studies in Dutch adolescents [12,45], our participants can be considered excessive non-active video gamers, which likely made it even more difficult to motivate them to become active video gamers. Further, it should be noted that our participants can not be described as inactive because the median physical activity was 10.5 hours per week. It could be that a more inactive group might be more susceptible for active games. The current results also indicate that the current commercially available active video games cannot (yet) truly compete with non-active video games because the intervention group participants told us that they preferred playing non-active video games and thought that the active video games were boring. This finding accords with those of our previous studies that indicated that turning non-active video gamers into active video gamer might be difficult to achieve [12, 22].

Other differences between our study and earlier studies that did find beneficial effects were included the targeting younger children (8–14 years) [27,29], the use of school setting [26] and the incorporation of active video games into a larger weight management program [29] in the earlier studies. Furthermore, all of the previous studies utilized shorter intervention and follow-up periods. Younger children (8–12) might be more receptive to active video games. Focus groups among 8 to 12 year-olds showed have indicated that these younger children are less critical about active video games and even prefer active video games above non-active video games [46]. Future studies should evaluate which strategies can increase sustainable active video game play and whether other target groups (e.g., younger children) are more suitable for active video game interventions.

These potential reasons for the lack of effects in the intervention group do not however explain the observation that the control group exhibited anthropometric improvements while the intervention group remained stable. Moreover, it is remarkable that the self-reported sedentary screen time of the intervention group was less than that of the control group, which might be related to social desirability bias (see limitations below).

During recruitment, we communicated that all participants would receive the Move video games (either at the start of the study or after ten months). Therefore, at the end of the study, the control group also received the active video games. If we had known the study results in advance, we would not have provided the active video games to the control group. Nevertheless, based on the intervention effects we observed (i.e., the anthropometrics data from the intervention group remained relatively stable over time and their self-reported sedentary screen time decreased over time), we do not believe that providing the active video games to the control group would have been harmful.

### Limitation and strengths

The limitations of the current study are the self-reported secondary outcome measures and their susceptibility to social desirability and recall biases. Furthermore, the active video game questionnaires and calendars were not checked for validity. The intervention group might have provided more socially desirable answers than the control group because, as part of the intervention, the participants in the intervention group were asked to replace non-active gaming with active gaming. Unfortunately, we could not use objective data to monitor physical activity and game behavior. Video game play data are stored in the game consoles, but due to privacy regulations these data could not be obtained.

The measured anthropometrics are clear strengths of the present study. In our analyses, we used BMI-SDS scores that were based on an external reference (i.e., the population level growth data [1]) as recommended by Must and Anderson (2006) [47]. BMI-SDS values provide insight into how a participant’s BMI is related to other individuals of the same age and sex within the general Dutch population. In other words, the BMI-SDS indicates the extent to which an individual’s BMI value deviates from ‘normal’ and is easier to interpret than crude BMI; therefore BMI-SDS values are used by most pediatricians in The Netherlands. Other strengths include the large number of participants, the low attrition and thus power of the study, the long intervention period, and the comprehensive process evaluation that provided valuable additional insights and information for the interpretations of the results.

### Recommendations for future research

Thus far, the evidence relating to the use of active video games as a weight management tool for overweight or obese children is mixed, and evidence for active gaming as a means to prevent excessive weight gain in normal-weight children is still lacking after the present study. Given the popularity of video gaming among youth, further high-quality research is needed regarding on how, when and among whom active video gaming can be effectively applied to promote health and prevent weight gain. Based on findings from earlier studies on the treatment of overweight and obesity, the integrating of active video games into broader health promotion programs seems most promising. Therefore, future studies should examine this issue further and focus on, for example, how to combine and target multiple settings, such as the home, school, and neighborhood, and examine the effectiveness of such integral multi-setting programs in the long-term. When evaluating active video game interventions, we recommend to use objective measurements (e.g. accelerometry) in combination with self-reports for assessing physical activity and sedentary behavior. Regarding assessing video gaming behavior, preferably the data that are electronically registered in the consoles should be used because this process would avoid participant burden and overcome the problems of under or over reporting and the providing of socially desirable answers. Therefore, it is important that the game industry make the stored game data available for research purposes. Also we recommend the use of objective measures of physical activity (e.g. accelerometry) in combination with self-report.

The process evaluation results indicated that there is a need for a new generation of active video games that are better able to compete with non-active games in terms of fun, attractiveness, persuasiveness and sustainability. Thus far, the majority of studies have focused on commercially available active games for consoles such as the Xbox 360 Kinect, PlayStation 3 Move and Nintendo Wii. In addition to these console-based active video games, one could also think of more mobile games on platforms such as smartphones or tablets. These platforms are suitable for the utilization of using technologies such as the Global Positioning System and augmented reality, which could increase the possibilities for physical activity in and beyond the game. Furthermore, the focus of research should not be limited to only active video games that are developed by the commercial entertainment game industry (as in this trial), whose main goal is to sell as many devices and video games as possible. Rather, future studies should also focus on active video games, such as the active game Olympus [48], that are based on behavioral theories and developed by serious game designers whose main goal is to promote physical activity. Furthermore, it is important that future studies also focus on the potentially undesirable effects of active video game interventions, such as reduced levels of prosocial behavior and life satisfaction and neglect of school responsibilities, because some studies have observed such effects to be associated with non-active video game play [49,50].

### Conclusion

Despite the finding that self-reported sedentary screen time was reduced, the current study suggests that providing active video games to a group of excessive non-active video gamers in their home environment is not effective and might even be counter-effective in altering anthropometrics. In conclusion, the present active video game intervention is not a suitable tool for the prevention of excessive weight gain among gaming adolescents.

## Supporting Information

S1 CONSORT Checklist (DOC)

S1 Protocol Trial Protocol. (PDF)

S1 Data File Anthropometrics. (SAV)

S2 Data File Questionnaires. (SAV)
